# The influence of objectives, learning experiences and examination blueprint on medical students' examination preparation

**DOI:** 10.1186/1472-6920-5-39

**Published:** 2005-12-16

**Authors:** K McLaughlin, S Coderre, W Woloschuk, T Lim, D Muruve, H Mandin

**Affiliations:** 1 Division of Nephrology, Foothills Hospital. 1403 29^th ^St. NW, T2N 2T9. Calgary, Alberta, Canada; 2 Department of Medicine, University of Calgary, Health Sciences Centre, 3330 Hospital Drive NW, T2N 4N1. Calgary, Alberta, Canada; 3 Faculty of Medicine (Program Evaluator), University of Calgary. Health Sciences Centre, 3330 Hospital Drive NW, T2N 4N1. Calgary, Alberta, Canada; 4 Faculty of Medicine, University of Calgary. Health Sciences Centre, 3330 Hospital Drive NW, T2N 4N1. Calgary, Alberta, Canada

## Abstract

**Background:**

The influence of intended and informal curricula on examination preparation has not been extensively studied. This study aims to firstly describe how students utilized components of intended and informal curricula to guide examination preparation, and secondly to study the relationship between examination preparation and performance.

**Methods:**

Students received a pre-examination questionnaire to identify components from the intended curriculum (objectives and examination blueprint), and informal curriculum (content emphasised during lectures and small groups), used during examination preparation. Multiple logistic regression was used to study the relationship between these variables and student performance (above versus at or below average).

**Results:**

Eighty-one students participated. There was no difference in the proportions using the examination blueprint, content emphasised during lectures, and content emphasised during small groups (87 – 93%) but fewer students used objectives (35%, p < 0.001). Objectives use was associated with reduced odds of above average examination performance (adjusted odds ratio 0.27 [0.07, 0.97], p = 0.04).

**Conclusion:**

When preparing for the renal course examination, students were influenced at least as much by the informal as the intended curriculum. Of the two intended curriculum components, the examination blueprint appeared to be more widely used than the course objectives. This decreased use of objectives on examination preparation did not appear to have a detrimental effect on student performance.

## Background

**Figure 1:**
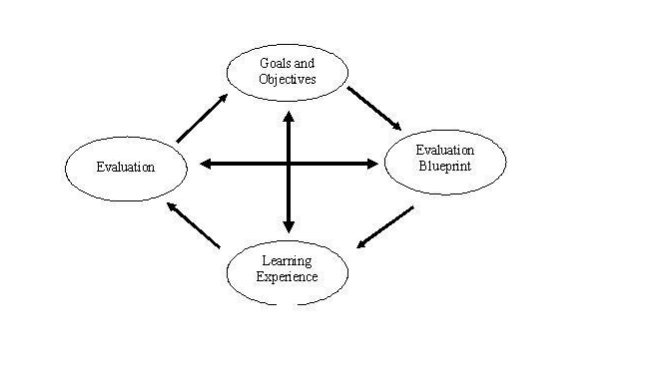
The Cybernetic Model.

Medical curricula are generally organized into three major pillars: objectives, learning experiences and evaluation [1]. While it is assumed that all three exert an influence on the process of learning, the relative contribution of each has not been studied [2,3]. All structured educational activities should, ideally, have a single curriculum that guides student learning – the 'intended curriculum' [4,5]. Hafferty defines this as "the stated, intended and formally offered and endorsed curriculum" [4]. This curriculum is best reflected by course objectives and the examination blueprint, linked to congruent learning experiences and evaluation as depicted by the 'cybernetic model' (figure 1). Any incongruence within these pillars of education may lead to the development of an alternative curriculum. For example, teaching that veers from the intended curriculum as identified by the course objectives may create a second curriculum – the 'informal curriculum' [4]. Hafferty defines this curriculum as "an unscripted, predominantly ad hoc, and highly interpersonal form of teaching and learning that takes place among and between faculty and students" [4]. The personal nature of content taught by faculty in lectures and small groups best reflects this definition of the informal curriculum. Hafferty also defines a third curriculum, the 'hidden curriculum' [4,6], as "a set of influences that function at the level of organizational structure and culture" [4]. Repeated discrepancy between what students perceive that they need to know [for examination purposes] and the stated course objectives can potentially lead to a 'local culture' whereby a hidden curriculum is created. The hidden curriculum highlights the importance of congruency within the cybernetic model.

In an attempt to share the intended curriculum with students and minimise the effect of alternative curricula, at the University of Calgary 'core documents' are given to students to provide emphasis on course material and to guide learning. In addition to course objectives, most core documents also include a detailed examination blueprint. This blueprint is used to guide both the selection of course content and the end of course examination [7]. The relative influence of the components of the intended curriculum, represented by course objectives and examination blueprint, on students' preparation for the end of course examination is unknown. Similarly, the influence of the informal curriculum, represented by content taught in lectures and small groups, on examination preparation is also unknown. Knowledge of which components influence examination performance would be valuable in increasing the efficiency and effectiveness of curricular design.

The first objective of this study was to describe the frequency with which students utilized components of the intended and informal curricula to guide preparation for the first year undergraduate renal course examination. This course has a total of 108 hours of instruction, with close to 50% of these hours spent in small group teaching that closely follows relevant lectures. The second objective was to study the relationship between the use of these components and examination performance. It is our belief that by publishing the examination blueprint, the effect of the hidden curriculum is reduced, such that the process of evaluation becomes an ally rather than an enemy of the intended curriculum. Hence, it was hypothesized that publication of the objectives in the core document (intended curriculum), including the examination blueprint, would lead to increased utilization of these components by the students in their examination preparation, and consequently increased success in the examination.

## Methods

On the day prior to the end-of-course certifying examination, first year medical students in the renal course were given a questionnaire that asked about their preparation for the upcoming exam. Students were asked to indicate, using a 5-point Likert type scale, the extent to which components from the intended curriculum (course objectives and the examination blueprint), and components from the informal curriculum (content emphasised during lectures and small groups), influenced their examination preparation. The responses for these four components were then dichotomised [8]. The positive responses from the scales ('agree' and 'strongly agree') were combined and given a value of 1. Negative responses were combined in a similar fashion and given a value of 0. Non-responses and neutral responses ('neither agree nor disagree') were coded as missing values.

To study the relationship of these four variables to student performance the students were divided into two groups, those with above average scores, and those with scores at or below average, on the renal end-of-course examination. Using the mean score to split the class instead of pass-fail was based upon the fact that the number of students failing was small and considering this as an end-point would reduce the power of the study. Assuming a sample size of 80 students, considering the students above average versus those at or below average, provided sufficient power (>0.80) to detect a difference of ≥ 32% in utilisation of any of these four examination preparation strategies between the two groups. Fisher's exact test was used for two-sample comparisons of proportions. The four potential influences on examination preparation were entered into a multiple logistic regression model that also considered two-variable interaction terms. The dependent variable was whether or not the students had above average examination scores, and the four individual components were considered as explanatory variables. A backward elimination procedure was used in which nested models were compared using the likelihood ratio test. All statistical tests were two-sided and a p value of <0.05 was considered statistically significant. All statistical analyses were performed using STATA 7.0 software [9].

## Results

Eighty-one of one hundred students in the class completed the questionnaire. For the end of course examination the class mean (± SD) was 83.92 (± 7.80)% and the reliability coefficient (Cronbach's alpha) was 0.74. The proportion of students using the individual components for examination preparation is shown in Table 2 and ranged from approximately 35% for objectives to 93% for content emphasised during small group sessions. There was no difference in the proportions of students using the examination blueprint, content emphasised during lectures, and content emphasised during small groups. The proportion of students using course objectives was, however, significantly lower than the other three combined (P < 0.001).

**Table 2:** Prevalence of variables influencing examination preparation

**Variable influencing examination preparation**	**% Students using this variable**
Objectives	34.7%*
Examination blueprint	86.7%
Content emphasised during lectures	92.5%
Content emphasised during small groups	93.0%

*Significantly lower than the other 3 groups combined, P < 0.001.

Comparing students above and those at or below the mean examination score, there was no difference in the proportions of students using the examination blueprint (85 versus 87.9%, p = 1.0), content emphasised during lectures (90.3 versus 94.3%, p = 0.7) and content emphasised during small groups (94.6 versus 90.3%, p = 0.6) to guide examination preparation. There was a trend towards reduced objectives use by above average students (22.2 versus 42.9%, p = 0.08). By multiple logistic regression there were no significant interactions between variables. The only variable associated with examination performance was the use of objectives, which was associated with reduced odds of having an above average score for this examination. These data are shown in Table 3.

**Table 3:** Relationship between variables influencing examination preparation and the odds of obtaining an above average examination score

**Variable influencing examination preparation**	Odds ratio of above average score* [95% CI]	**P value**
Objectives	0.27 [0.07, 0.97]	0.04
Examination blueprint	0.95 [0.20, 4.52]	0.95
Content emphasised during lectures	0.62 [0.04, 9.61]	0.73
Content emphasised during small groups	5.74 [0.39, 83.16]	0.20

*The odds ratio for each variable is adjusted for the effects of the other three variables in the model

## Discussion

The first objective of this study was to describe the relative influence of components of the intended and informal curricula on examination preparation in a group of first year medical students. As demonstrated in Table 2, course objectives were used by a significantly smaller proportion of students to guide examination preparation than the evaluation blueprint, content emphasised during lectures and material emphasised during small groups. The second objective was to study the relationship between the use of components of the intended and informal curricula and examination performance. We found, in Table 3, that the use of course objectives to guide examination preparation was associated with reduced odds of above average performance (or increased odds of below average performance), while use of the other three components were not significantly associated with students' performance.

Given the emphasis placed on the creation of objectives at all levels of education, the finding that course objectives were downplayed by students when preparing for examinations was unexpected. Several possible explanations for this were considered, the first being that the objectives were incongruent with the evaluation process. This was considered unlikely as the objectives and the final examination were matched using an examination blueprint. Nevertheless, to address this possibility, an analysis was performed on the information from the end-of-course questionnaire, on which students provide feedback about both the course and the certifying examination. Of the 61 (out of 100) students who responded to the statement, "the final evaluation reflected the course objectives" there were none that disagreed with the statement, suggesting objectives were not disregarded due to incongruence with the examination. Note, however, that while 61 students responded to this statement, only 28 actually used objectives.

The second explanation considered for the low utilization of objectives was that the objectives were incongruent with the material actually taught. To address this, all the course preceptors (who were given the list of course objectives prior to the course) were asked whether they used the course objectives as a guide in the preparation of the learning experiences. Of the 19 (out of 32) preceptors who responded, 16 (84%) said that they were aware of the objectives, although only 7 (37%) prepared their learning experiences based on the objectives. The low frequency of preceptors utilizing course objectives in their preparation for lectures and small groups creates the potential for an informal curriculum. Despite course objectives also being downplayed by preceptors, there appeared to be congruence between the learning experiences and the evaluation process as only 4% of students disagreed with the statement that "the exam tested material actually taught" and 2% disagreed with the statement that "the exam tested important aspects of subject matter". The observation of congruence between the material taught and the evaluation process suggests that the informal (or delivered) curriculum did not significantly veer from the path of the intended curriculum, i.e., the material taught matched the course objectives.

The third explanation considered for the low utilization of objectives was that they were poorly written. This was considered an unlikely explanation as the course objectives were essentially the same as those adopted by the Medical Council of Canada and written by the same person who has considerable expertise in this area [10].

Having failed to demonstrate incongruence between objectives and the other pillars of education, it can be concluded that the most likely explanation for objectives being disregarded was that they had become redundant in the face of other 'stronger' influences on examination preparation. As 'actions speak louder then words' it is not surprising that the proportion of students using components of the informal curriculum was greater than the proportion using objectives on examination preparation (which is not a problem if these are congruent). It is interesting to note, however, that the proportion of students using the examination blueprint was not different from the proportion using components of the informal curriculum. This observation would suggest that publishing the examination blueprint may be a better way of guiding learning by the intended curriculum than publishing the course objectives.

The second finding of the paper was an apparent negative impact of using objectives to guide examination preparation. The explanation for this is not immediately clear. As discussed above, the observed congruence between objectives and the evaluation process would suggest that the objectives themselves were not misleading. As such, it can be concluded that the more likely explanation for the association between the use of objectives and lower (or equal) than average performance was reverse causality. A possible interpretation of these is that most students did not use objectives in examination preparation because they didn't need to use them. Weaker students, on the other hand, may still have been unsure as to what they should have learned by the end of the course. Using objectives at this late stage might, therefore, be an indicator, rather than a cause, of poor examination preparation.

There are some important limitations to this study that should be considered. The study sample was a single class during a single undergraduate course and, as such, the findings may not be generalizable. Another important consideration is the fact that not all courses have an examination blueprint and it is possible, if not probable, that objectives have a greater influence on examination preparation when they represent the only published component of the intended curriculum. Similarly, the congruence between the material taught and the evaluation process may have afforded students the luxury of disregarding the objectives, which may not be the case if the informal curriculum veers significantly from the intended curriculum. Lastly, this study was limited in that exit interviews or focus groups were not conducted to explain why students did not turn to the objectives as much for guidance. These sorts of investigations would be of clear benefit in future similar studies.

## Conclusion

The results observed in this study suggest that in preparing for the renal course examination, students appeared to be influenced at least as much by features of the informal curriculum (content in small groups and lectures) as by features of the intended curriculum (course objectives and examination blueprinting). This is an important reminder to medical educators that, once the intended curriculum is set, careful attention is required to the informal curriculum for the initial objectives to be met. With regards to the components of the intended curriculum, the examination blueprint was used by the majority of students whereas a minority employed objectives to guide examination preparation. The strategy of disregarding objectives in examination preparation appeared to be effective, suggesting that well prepared students had no need to review the objectives at this stage. This is also key in course preparation, in highlighting the importance and potential power of an examination blueprint.

We would guard against concluding from these results that objectives are redundant, as we do believe that all worthwhile educational experiences are initially driven by objectives. Instructional objectives serve two important purposes: to guide course design and assessment, and secondly to communicate expectations to students. It may be that in the final preparatory stage for examinations, when these two purposes have already been served, students turn their attention to other important features of a course, such as the examination blueprint. The notion that the more successful students were able to disregard objectives when preparing for their examination may be a testimony to the congruence between the pillars of education in a well-designed course.

## Competing interests

The author(s) declare that they have no competing interests.

## Authors' contributions

- KM conceived of the study, participated in its design, coordination and final manuscript draft

- SC participated in manuscript revisions, and drafted the final manuscript

- DM and TL participated in study design and coordination

- HM participated in a detailed revision of the initial manuscript draft

- WW participated in the study/survey design, and manuscript drafting

**Table 1:** The renal course blueprint

**Presentation**	**Number of questions**	**Diagnosis**	**Investigation**	**Treatment**	**Basic Science**
Hypernatremia	2	1	0	0	1
Hyponatremia	6	3	1	1	1
Hyperkalemia	6	3	1	1	1
Hypokalemia/Hypomag.	4	2	0	1	1
Acidosis	8	4	1	1	2
Alkalosis	4	2	0	1	1
ARF	8	5	2	1	0
CRF	8	5	1	1	1
Hematuria	4	3	1	0	0
Proteinuria	6	3	0	0	3
Edema	3	1	0	1	1
Scrotal mass	4	2	1	1	0
Retention	2	1	1	0	0
Hypertension	8	4	2	2	0
Polyuria	2	1	0	0	1
Renal colic	4	2	1	0	1
Dysuria	2	1	1	0	0
Incontinence	2	2	0	0	0
TOTAL	83	45	13	11	14

## Pre-publication history

The pre-publication history for this paper can be accessed here:


